# Magnetic resonance and diffusion tensor imaging of the adolescent rabbit growth plate of the knee

**DOI:** 10.1002/mrm.29432

**Published:** 2022-09-15

**Authors:** Ola Kvist, Peter Damberg, Zelong Dou, Johan Sanmartin Berglund, Carl‐Erik Flodmark, Ola Nilsson, Sandra Diaz

**Affiliations:** ^1^ Department of Paediatric Radiology Karolinska University Hospital Stockholm Sweden; ^2^ Department of Women's and Children's Health Karolinska Institute Stockholm Sweden; ^3^ Department of Clinical Neuroscience Karolinska Institutet Stockholm Sweden; ^4^ Department of Health Blekinge Institute of Technology Karlskrona Sweden; ^5^ Department of Clinical Sciences in Malmö Lunds University Lund Sweden; ^6^ School of Medical Sciences Örebro University Örebro Sweden; ^7^ Department of Radiology Lunds University Lund Sweden

**Keywords:** cartilage, diffusion‐tensor imaging (DTI), growth plate, magnetic resonance imaging (MRI), skeletal maturation

## Abstract

**Purpose:**

To assess the ability of MRI‐DTI to evaluate growth plate morphology and activity compared with that of histomorphometry and micro‐CT in rabbits.

**Methods:**

The hind limbs of female rabbits aged 16, 20, and 24 wk (n = 4 per age group) were studied using a 9.4T MRI scanner with a multi‐gradient echo 3D sequence and DTI in 14 directions (*b*‐value = 984 s/mm^2^). After MRI, the right and left hind limb were processed for histological analysis and micro‐CT, respectively. The Wilcoxon signed‐rank test was used to evaluate the height and volume of the growth plate. Intraclass correlation and Pearson correlation coefficient were used to evaluate the association between DTI metrics and age.

**Results:**

The growth plate height and volume were similar for all modalities at each time point and age. Age was correlated with all tractography and DTI metrics in both the femur and tibia. A correlation was also observed between all the metrics at both sites. Tract number and volume declined with age; however, tract length did not show any changes. The fractional anisotropy color map showed lateral diffusion centrally in the growth plate and perpendicular diffusion in the hypertrophic zone, as verified by histology and micro‐CT.

**Conclusion:**

MRI‐DTI may be useful for evaluating the growth plates.

**Abbreviations**
AD: axial diffusivity
FA: fractional anisotropy
MD: mean diffusivity
μCT: micro‐CT
R: radial diffusivity

## 1 INTRODUCTION

Longitudinal growth occurs at the growth plate, a cartilaginous structure located between the metaphysis and epiphysis of long bones. As a final step in the maturation process, the growth plate undergoes epiphyseal fusion.^1^ In humans and some mammals, including rabbits, epiphyseal fusion occurs during sexual maturation.^2^, ^3^ Therefore, unlike in mice or rats, the similarity between the skeletal maturation process of rabbits and humans makes the rabbit a good model for simulating a human adolescent growth plate, particularly for evaluating the closure of the growth plate.^4^

Microscopic evaluation of tissue provides information about its cellular structure and properties. Therefore, even though histology entails an invasive procedure, it is considered the reference standard for the assessment of a given tissue. The growth plate matures over time until it becomes part of the mature bone. Therefore, radiological studies of the growth plate should be stratified according to age and validated by comparison with the reference standard, that is, histology.

Various imaging modalities have been used to assess the growth plate and skeletal maturation. The most commonly used method is ionizing radiation with an x‐ray of the left hand and wrist, and less frequently, CT.^5^, ^6^, ^7^, ^8^, ^9^ Micro‐CT (μCT) is a high‐resolution method for evaluating bones both in vivo and ex vivo.^10^ MRI uses various sequences, such as spoiled gradient recalled echo sequence (SPGR), proton density (PD), and T2 sequences, to visualize different tissues and structures, including the cartilaginous growth plate.^11^ These sequences are most commonly used to evaluate articular cartilage and less often to assess growth plate cartilage. Radiological studies, both with ionizing radiation and MRI, have been performed in forensic medicine using subjective evaluations without direct comparisons with tissue samples.^9^, ^12^, ^13^

DTI is a more recent method used to visualize regions with directionally restricted diffusion of water molecules and to evaluate microstructures, such as muscles and peripheral nerves.^14^, ^15^ Chondrocytes in the growth plate have a columnar orientation with anisotropic properties.^16^ Further, it has been observed that these columns extend into the metaphysis and can be visualized using DTI.^17^ However, few studies have addressed this hypothesis in animals^18^, ^19^, ^20^ or humans.^17^, ^21^, ^22^, ^23^

Methods that allow visualization of growth plate cartilage with improved resolution would be clinically useful in the assessment of congenital and acquired disorders, such as skeletal dysplasia's, infectious and inflammatory diseases, and Salter–Harris fractures.^24^ Potentially, they can also be used in bone age assessments and adult height prediction models.

The aim of this study was to use MRI settings that can be clinically applied to validate MRI findings in the growth plate of rabbits of different ages compared to histomorphometry and μCT. A secondary aim was to link MRI‐DTI findings with histology, thereby proving that DTI depicted the activity of the growth plate.

## 2 METHODS

### 2.1 Animals

The study was approved by the Regional Animal Ethics Committee (permit no. 14436–2019). Twelve female New Zealand white rabbits were used in the present study. The rabbits were stratified into three age groups (16, 20, and 24 wk old, four in each age group) to simulate adolescence in girls.^25^ The rabbits were premedicated with a subcutaneous injection of medetomidine 0.5 mg/kg and euthanized with an overdose of pentobarbital administered through the lateral auricular vein. The hind limbs were separated from the body prior to imaging to improve the image quality and were imaged immediately after euthanasia.

### 2.2 MR imaging

All MRI acquisitions were made at a temperature of 25°C. MRI data were acquired using a 9.4T preclinical MRI scanner (Varian MRI system, Agilent Technologies, Palo Alto, CA, USA) running VnmrJ 4.2 equipped with a gradient insert with an inner diameter of 12 cm capable of generating gradient fields of 60 mT/m. An actively tuned circularly polarized birdcage coil with an inner diameter of 72 mm (Rapid Biomedical GmbH, Rimpar, Germany) was used for excitation, and a four‐channel phased array coil in a semicircular housing with an inner diameter of 50 mm (Rapid Biomedical GmbH, Rimpar, Germany), originally designed for rat heart applications, was used for signal reception.

The left and right hind limbs from one rabbit were arranged side by side using rubber bands and tape, with the knee joints in extended positions. The hind limbs were secured in the prone position in the four‐channel phased array coil with the long axis of the limbs parallel to the magnetic field and the patella facing the sensitive region at the bottom of the receiver coil.

The cartilage in the growth plate was visualized using a multi‐gradient echo 3D sequence with the following relevant parameters: FOV 60 × 60 × 60 mm^3^, matrix 256 × 256 × 256; this translated to a voxel size of 234 μm. The readout gradient was parallel to the magnetic field, flip angle 30°, recovery time 30 ms, and echo times 1.46, 3.74, 6.22, and 8.60 ms with 16 dummy scans. The total acquisition time for the multigradient echo 3D sequence was 30 min and 4 s.

DTI was acquired using a diffusion‐weighted spin‐echo sequence with the following relevant parameters: 28 slices, no gap, 1 mm slice thickness with vertical slice‐select direction, that is, from the patella to the hollow of the knee, TE 25.6 ms, recovery time 2.5 s, FOV 90 × 60 mm^2^, matrix 256 × 128, which translated to an in‐plane resolution of 350 × 460 μm. The readout gradient was parallel to the magnetic field and the phase‐encode direction from left to right with four dummy scans. The diffusion encoding and decoding gradients were 4 ms long with an amplitude of 15.93 mT/m separated by 16 ms resulting in a *b*‐value of 984 s/mm^2^. DTI images were acquired along 14 diffusion directions in addition to two reference images. The total acquisition time for the DTI sequence was 1 h, 15 min, and 50 s.

### 2.3 MRI image segmentation and analysis

The growth plates were manually traced by a pediatric radiologist with 6 y of experience (O.K.) using the ITK Snap software version 3.8.0 (http://itksnap.org) on the multi‐gradient echo 3D sequence. The volume of the growth plate was then calculated. In the coronal plane, the central part of the growth plate was selected, and the growth plate was divided into 10 different portions. The height of each portion of the growth plate was measured and averaged. To calculate the DTI metrics and extract the tractography, DSI Studio (http://dsi‐studio.labsolver.org) was used, and every region of interest was manually traced. The analysis was performed by two pediatric radiologists with six (O.K.) and 16 y (S.D.R.) of experience in pediatric radiology. The diffusion tensor fractional anisotropy (FA), mean diffusivity (MD), axial diffusivity (AD), and radial diffusivity (RD) were acquired from the traced regions.

**FIGURE 1:**
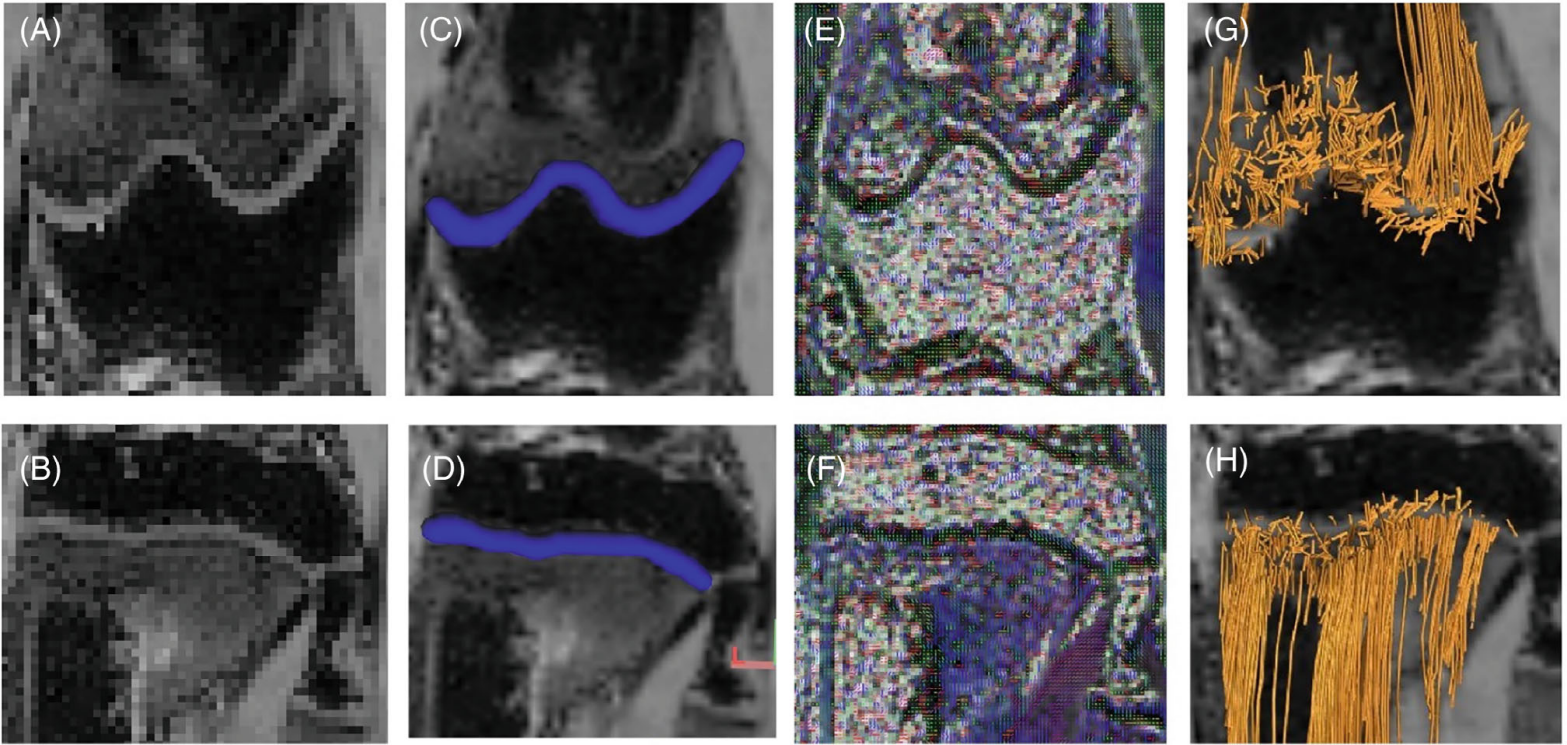
Image segmentation regarding DTI. The growth plate is segmented manually from a coronal B0 image of the femur (A) and the tibia (B) of a 16‐wk‐old rabbit. The region of interest is visualized in blue on a multi‐gradient echo 3D sequence in both the femur (C) and tibia (D). The fractional anisotropy chart on both locations (E and F) shows the orientation of the diffusion in every single voxel. DSI studio software generates tracts from the region of interest that are visualized in the femur (G) and tibia (H). From these tracts, statistics about tract volume, length, and number of tracts, as well as the different DTI values (FA value, MD, AD and RD), can be obtained. The colors depicted in each voxel on the FA map correspond with the direction of the main vector. (Blue: craniocaudal movement; red: lateral movement; green: antero‐posterior movement)

On coronal images, the diffusion direction of each voxel was visualized, and regions of interest and tractographies of the growth plates were created (Figure 1). The colors depicted in each voxel on the FA map correspond to the direction of the main vector (blue: craniocaudal movement; red: lateral movement; green: antero‐posterior movement). Based on previous research, a fiber tracking algorithm with a minimum FA threshold of 0.04 and a maximum turning angle of 45° between two adjacent voxels was used.^19^ All tracts were included in tractography, resulting in an acquired number of tracts, tract length, and volume. To rule out software malfunction, the raw data was reevaluated using the Diffusion Toolkit version 0.6.4 (trackvis.org, Martinos Center for Biomedical Imaging, Massachusetts General Hospital, Boston, MA).

### 2.4 Histological staining

After MRI, the right femur and tibia were cut at the front with a bone saw and then fixed in 4% paraformaldehyde at room temperature for 48 h, followed by decalcification with buffered 15% ethylenediaminetetraacetic acid for 4–6 wk at 4°C. Decalcified tissues were dehydrated and embedded in paraffin. A series of 6 μm paraffin sections from the distal femur and proximal tibia blocks were acquired with a microtome (Thermo Fisher Scientific, Waltham, MA, USA) and mounted onto TruBond 380 (Electron Microscopy Sciences, Hatfield, PA, USA) in a water bath, followed by drying on a warming plate. Slides were baked at 65°C for 60 min and deparaffinized with xylene and ethanol series (100%, 100%, 95%, 70%) before staining with Masson trichrome (HT15‐1KT, Sigma‐Aldrich, Heatherhouse, United Kingdom), according to the manufacturer's instructions.

### 2.5 Histomorphometry

High‐resolution images were obtained with a panoramic digital slide scanner (3DHISTECH Ltd, Budapest, Hungary), and growth plate height measurements were performed using compatible CaseViewer software (3DHISTECH). The measurements were performed by a PhD student (Z.D.) in medical science with 9 years of expertise in cellular biology. Specifically, measuring lines were drawn from the interface between the secondary ossification center and the growth plate cartilage to the interface between the growth plate cartilage and the metaphysis, where mineralization begins. In the central part of the growth plate, a coronal section was selected, and measurements were performed parallel to the chondrocyte columns and perpendicular to the interface borderlines. Ten individual measurements were taken for each growth plate and then averaged.

### 2.6 μCT

After MRI imaging, the left hind limb was used for μCT. The left hind limb was fixed with 70% EtOH and stored at 4°C until scanning. The μCT images were acquired using an x‐ray microscope (XRM) and Zeiss Xradia Versa 520 lab‐based scanner (Carl Zeiss X‐ray Microscopy, Pleasanton, CA, USA). The settings used to collect images included the emission source set at 80 kV and 7 W. An LE4 (low energy 4 filter) to reduce beam hardening artifacts. The samples were placed 3.8 mm from the source and 8.9 mm from the detector. A 0.4× objective was used, resulting in an field of view of 20.9 mm × 20.9 mm; 1024 × 1024 pixels per image translated to a 20 μm voxel size. A total of 801 images were taken at an exposure time of 2 s and step size of 0.45°, resulting in an acquisition time of 40 min and 32 s. Object Research Systems Dragonfly (Montreal, Canada) was used to identify the non‐mineralized growth plate, define it as a region of interest, and calculate its volume. In the central part of the growth plate, a coronal section was selected, and ten individual measurements were taken for each growth plate and averaged.

### 2.7 Statistical analysis

Wilcoxon signed‐rank tests were performed to compare growth plate volumes between MRI and μCT; the height of the growth plate between MRI, histomorphometry, and μCT; and diffusion metrics between the femoral and tibial growth plates. The Wilcoxon signed‐rank test was chosen because of the small sample size and assumption that the data were not normally distributed. Bland–Altman analysis for the paired measurements of growth plate height and volume was also performed. Intraclass correlation coefficients (ICC) (95% confidence interval) were used to measure inter‐observer agreement. ICC estimates and 95% confidence intervals were calculated as a two‐way random‐effects model. Statistical significance was set at *p‐*value <0.05. The ICC values were interpreted as follows: <0.5, poor agreement; 0.5–0.75, moderate agreement; 0.75–0.9, good agreement; and 0.9–1.0, excellent agreement.

Pearson's correlation coefficient was used to determine correlations between age, diffusion tensor values (FA, MD, AD, and RD), tract height, and volume. All statistical analyses were performed using SPSS Statistics v. 28.0 (IBM, Armonk, NY, USA). A *p*‐value <0.05 was considered significant.

## 3 RESULTS

### 3.1 Morphometry

**FIGURE 2:**
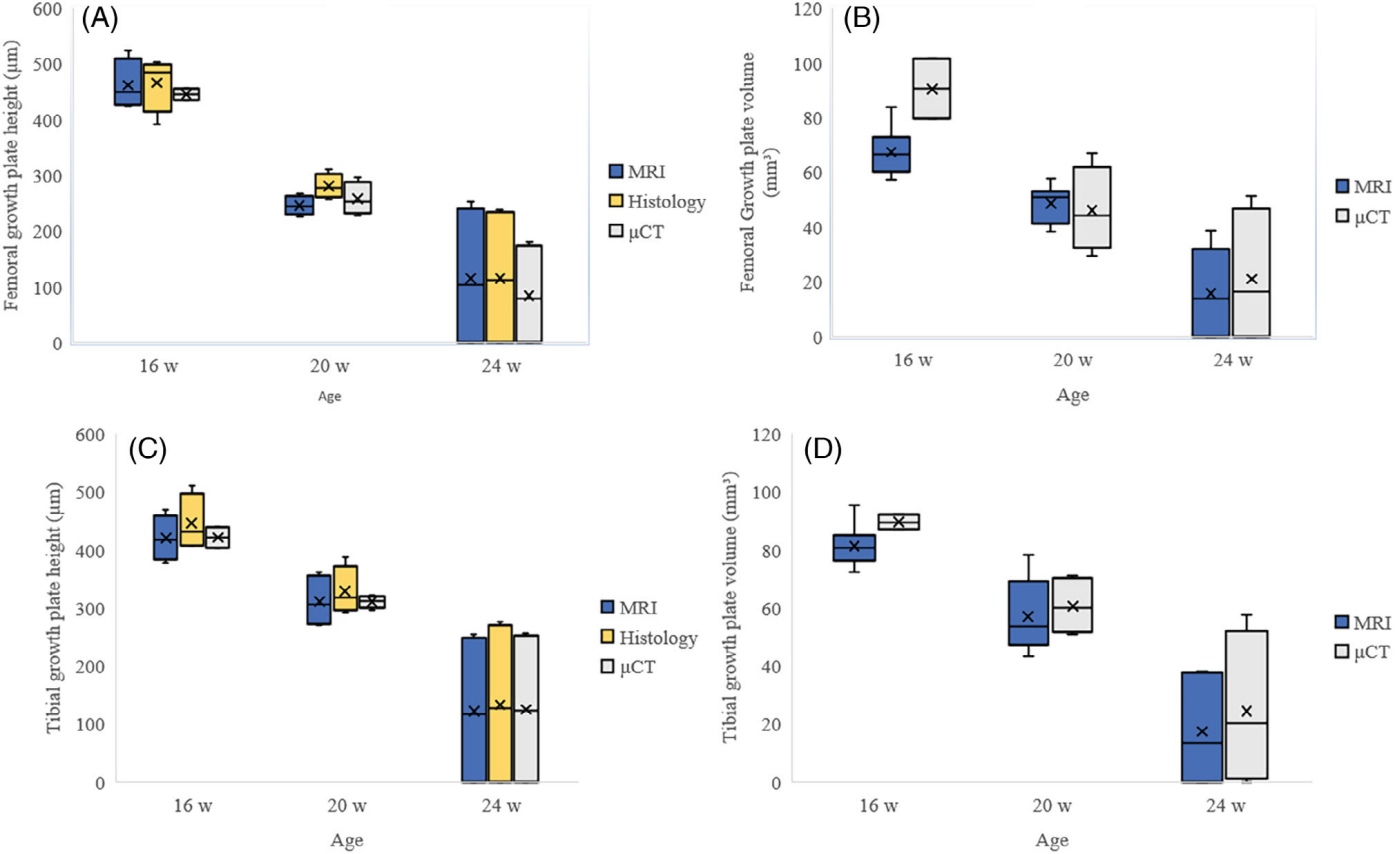
Demographic data regarding growth plate volume and height. The boxplots in the left column (A, C) compare the different heights on MRI, histomorphometry, and μCT. Those in the right column (B, D) compare the growth plate volumes on MRI and μCT. The upper row (A, B) are measurements for the femur and the lower (C, D) for the tibia (Volume is measured in mm^3^, and height is measured in μm)

**FIGURE 3:**
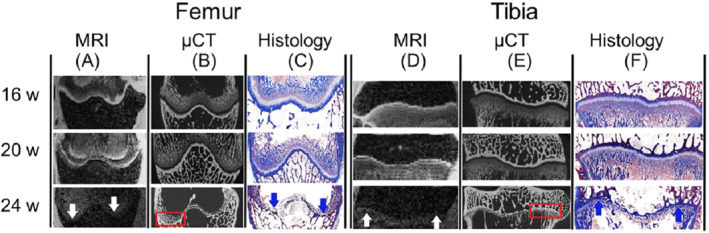
Coronal cross‐section of the growth plate in multi‐gradient echo 3D sequence on MRI, μCT, and histology (Masson's trichrome staining) in each age group. The multi‐gradient echo 3D sequence on MRI shows a high signal in the femoral (A) as well as the tibial (D) growth plate in the 16‐wk‐old rabbit; however, in the 24‐wk‐old, the growth plate is non‐detectable (white arrows). μCT shows a low‐attenuated growth plate in both the femur (B) and tibia (E). In the 20‐wk‐old rabbit, small bone bridging can be observed, and in the 24‐wk‐old rabbit, a completely fused growth plate is seen (red boxes). Histological representation shows the growth plate as a light blue line in 16‐ and 20‐wk‐old rabbits in both the femur (C) and tibia (F). The light blue growth plate line cannot be seen in the 24‐wk‐old rabbit; instead, a dark blue sclerotic remnant of the growth plate can be seen, which is sometimes referred to as a physeal scar (blue arrows)

**FIGURE 4:**
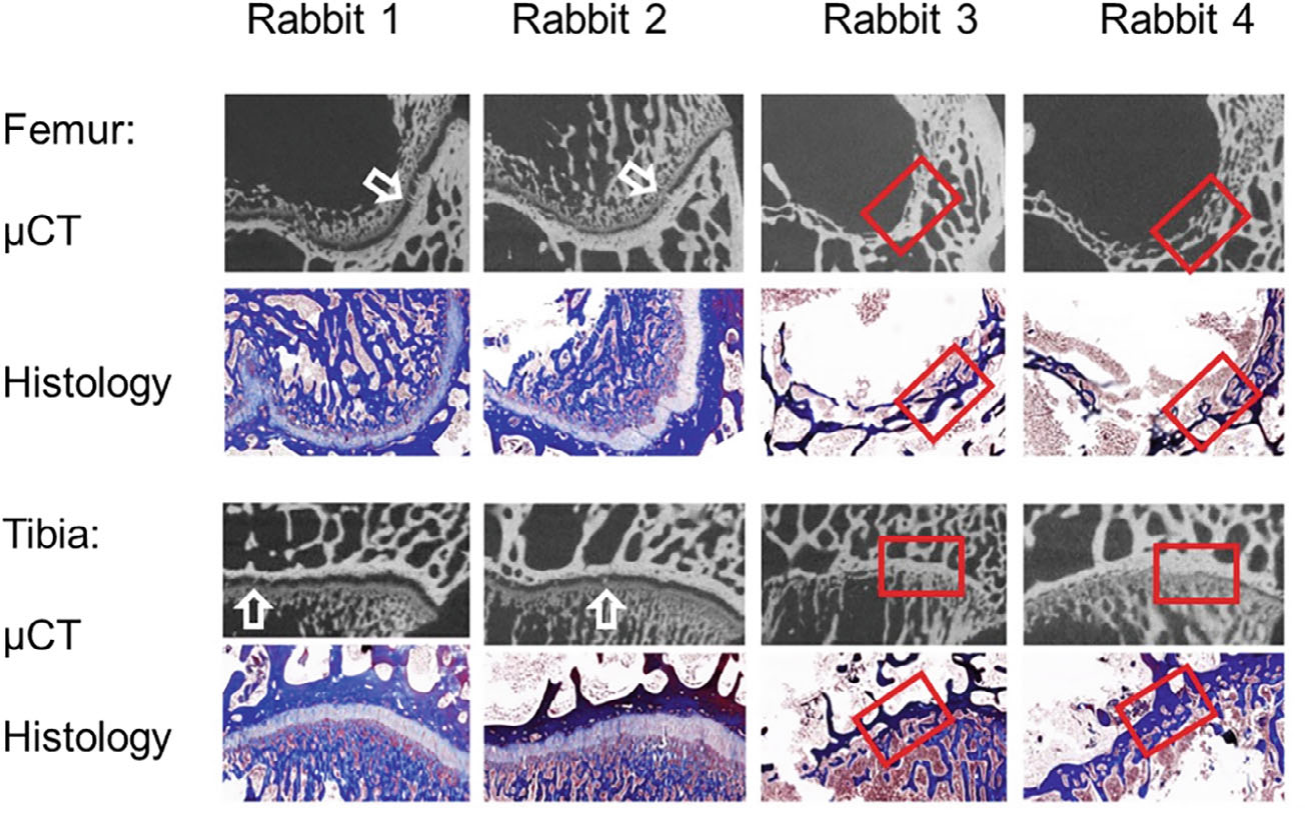
Representation of closure of the growth plate in 24‐wk‐old rabbits. Rabbits 1 and 2, demonstrating the beginning of closure of the growth plate with minor bone bridging on μCT (white arrow), which otherwise appears as an open growth plate on both histomorphometry and μCT. In contrast, in Rabbits 3 and 4, completely closed growth plate in both the femur and tibia on μCT as well as histomorphometry can be seen (red box)

**FIGURE 5:**
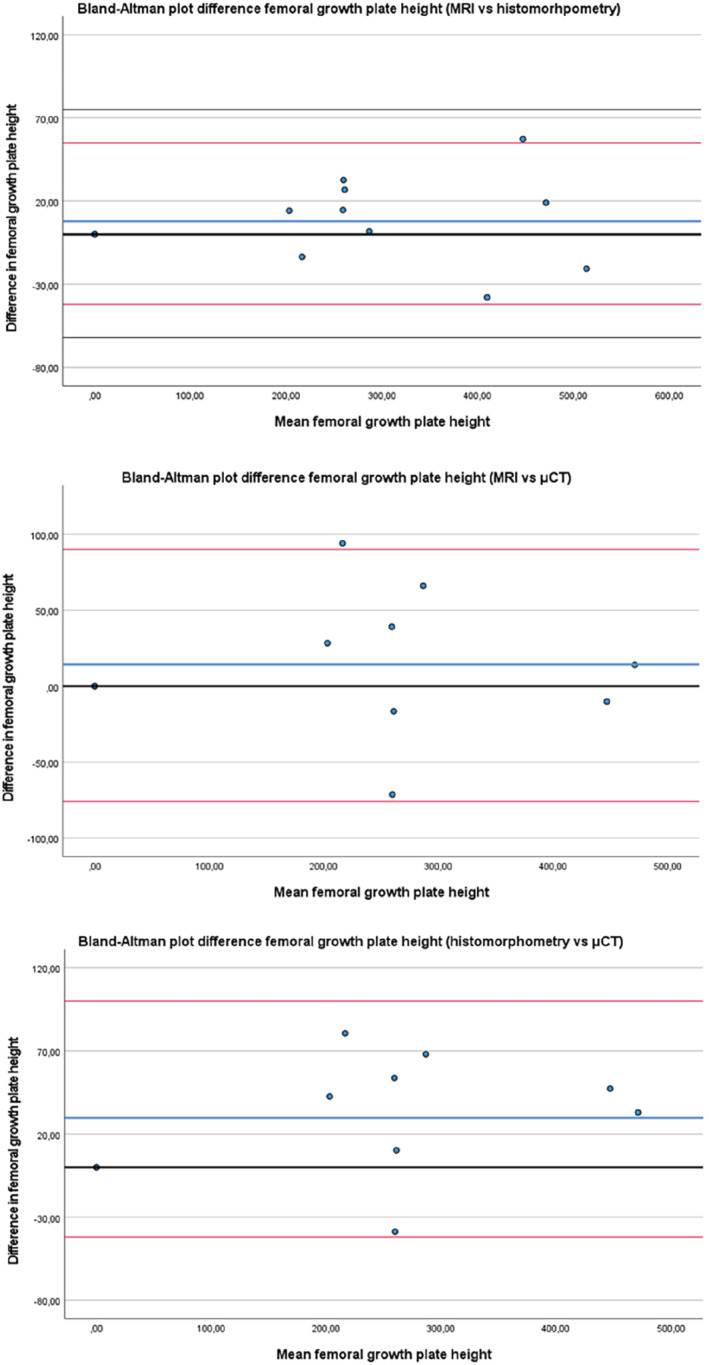
Bland–Altman plots regarding difference in the height measurements in the femur between MRI, μCT and histomorphometry. The bold blue line is the mean value and the red lines indicate the 95% confidence interval. (The zero value is indicated by a black line)

As expected, the height and volume of both the distal femur and proximal tibial growth plates declined with age in all modalities (Figure 2). The maturation process was visible in both the distal femur and the proximal tibia (Figure 3). In the 24‐wk age group, epiphyseal fusion occurred in both the distal femur and proximal tibia in two of the four animals (Figure 4). The Wilcoxon signed‐rank test did not show any statistical difference between the height and volumetric measurements in either the femur or tibia. The Bland–Altman plots showed that there was no significant difference between the modalities regarding the height measurements (Figure 5).

### 3.2 DTI

**FIGURE 6:**
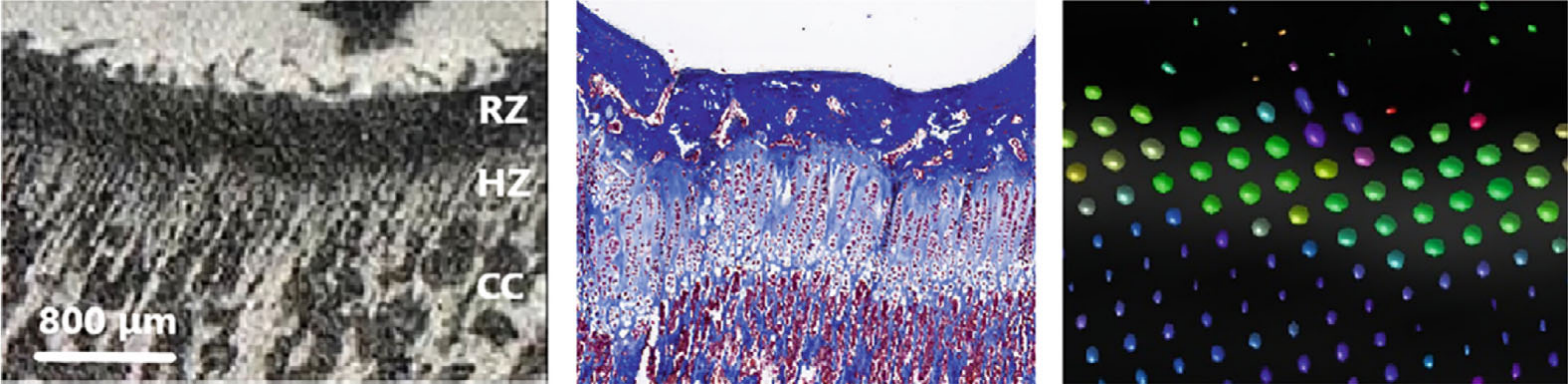
All images represent the same region of the tibial growth plate in a 16‐wk‐old rabbit. A, Close‐up of the growth plate on μCT (10 μm voxel size) shows a brush‐like appearance of bone trabeculae columns in the hypertrophic zone and its transtition to the metaphysis. B, Histologic sample with Masson's trichrome staining reveals columns of enlarged chondrocytes in the same region. C, Corresponding image and representative voxels in MRtrix3 software (MRView*)* help visualize the fractional anisotropy in each voxel, which is represented by a glyph. The glyph is an ellipsoid, and its size is related to the rate of diffusion. The shape of the glyph depends on whether the diffusion is isotropic (spherical glyph) or anisotropic (zeppelin‐shaped). The color is related to the direction of diffusion, as seen in a colored FA map. The diffusion is oriented in a cranio‐caudal direction on the border between the growth plate and the metaphysis, similar to the findings on μCT and histology. The diffusion pattern in the growth plate, on the other hand, is mostly almost isotropic, with diffusion following the orientation of the collagen II fibers. HZ, hypertrophic zone; RZ, resting zone

The colored FA map showed that the diffusion was sideways in the central portion (proliferative zone) of the growth plate but appeared to be perpendicular to the growth plate in the transitional zone between the growth plate and the metaphysis. High‐resolution images of the growth plate on μCT showed a brush‐like appearance of the transitional zone between the growth plate and the metaphysis (Figure 6). The histological samples verified that the brush‐like structure was a hypertrophic zone caused by the process of osteogenesis with columnar hypertrophic chondrocytes.^26^ Further assessment using MRtrix3 software (http://mrtrix.org *)* to visualize the FA in each voxel showed that there was a lower but anisotropic diffusion in a craniocaudal direction in the border between the growth plate and the metaphysis, like the findings of μCT and histology.

The tract number and volume were highest in the 16‐wk‐old group in both the femur (135.3 ± 23.9 mm^3^) and tibia (185.8 ± 47.1 mm3) and decreased over time (Table 1.). In contrast, tract length showed a different pattern, with the greatest tract length observed in the 20‐wk‐old group. MD, AD, and RD values declined with age, whereas the FA value increased. The results from the Diffusion Toolkit yielded comparable results.

**TABLE 1:** Tract‐based values from the growth plates measured on the DTI sequence

Anatomical location	Age (wk)	Tract number	Tract length (mm)	Tract volume (mm^3^)	FA value	MD (10^−3^ mm^2^/s)	AD (10^−3^ mm^2^/s)	RD (10^−3^ mm^2^/s)
Femur	16	2883.1 ± 337.4	2.99 ± 0.75	135.3 ± 23.9	0.20 ± 0.04	1.11 ± 0.07	1.31 ± 0.12	1.01 ± 0.06
	20	1693.0 ± 481.9	3.23 ± 1.23	78.6 ± 26.9	0.27 ± 0.07	0.70 ± 0.16	0.88 ± 0.19	0.61 ± 0.15
	24	1235.5 ± 800.0	3.13 ± 0.66	58.6 ± 39.4	0.29 ± 0.06	0.56 ± 0.18	0.70 ± 0.20	0.49 ± 0.17
Tibia	16	2864.0 ± 349.7	7.70 ± 3.14	185.8 ± 47.1	0.15 ± 0.03	0.93 ± 0.10	1.06 ± 0.10	0.87 ± 0.10
	20	2281.1 ± 759.0	9.77 ± 7.00	155.0 ± 91.8	0.21 ± 0.08	0.67 ± 0.14	0.80 ± 0.14	0.60 ± 0.14
	24	1123.4 ± 907.9	6.10 ± 5.34	60.6 ± 53.4	0.31 ± 0.08	0.57 ± 0.22	0.75 ± 0.25	0.49 ± 0.20

*Note*: All data are shown as mean and SD. The DTI metrics (FA, MD, AD, and RD) have a scalar value, with 0 indicating that the diffusion is isotropic and 1 indicating that diffusion only occurs in one direction (and is fully restricted in all other directions).

The Wilcoxon signed‐rank test indicated that tract length and volume were significantly greater in the tibia of 16‐ and 20‐wk‐old rabbits than in the femur. In addition, it showed that the DTI metrics (FA, MD, AD, and RD) were significantly lower in the tibial growth plate in 16‐wk‐old rabbits than in the femoral growth plate (Table 2).

**TABLE 2:** Wilcoxon signed‐rank test to evaluate whether there is a significant difference between the tibial and femoral growth plate

Age (wk)	Tract number	Tract length (mm)	Tract volume (mm^3^)	FA value	MD (10^−3^ mm^2^/s)	AD (10^−3^ mm^2^/s)	RD (10^−3^ mm^2^/s)
16	*Z* = −0.56 *p* < 0.58	*Z* = −2.52 *p* < 0.01	*Z* = −2.38 *p* < 0.02	*Z* = −2.52 *p* < 0.01	*Z* = 2.52 *p* < 0.01	*Z* = 2.52 *p* < 0.01	*Z* = −2.52 *p* < 0.01
20	*Z* = −1.54 *p* < 0.13	*Z* = −2.52 *p* < 0.01	*Z* = −1.96 *p* < 0.05	*Z* = −1.82 *p* < 0.07	*Z* = 1.26 *p* < 0.21	*Z* = −2.10 *p* < 0.04	*Z* = −0.14 *p* < 0.89
24	*Z* = −0.70 *p* < 0.48	*Z* = −1.68 *p* < 0.09	*Z* = −0.28 *p* < 0.78	*Z* = −0.42 *p* < 0.67	*Z* = 0.85 *p* < 0.40	*Z* = −1.26 *p* < 0.21	*Z* = −0.68 *p* < 0.50

*Note*: The test showed that the tract length and volume are greater in the tibial growth plate than in the femoral growth plate in 16‐ and 20‐wk‐old rabbits. In the 16‐wk‐old rabbits, significantly lower DTI metrics (FA, MD, AD, and RD) are seen in the tibial growth plate in comparison with the femoral growth plate. In the 20‐wwkeek‐old rabbits, a significant difference is observed in the AD value between the tibial and femoral growth plate. No significant difference is observed for any DTI metrics in the 24‐wk‐old rabbits. *Z* equals the sum of signed ranks divided by the square root of the sum squares.

The inter‐observer agreement (ICC) was excellent for the femur and good for the tibia regarding the MD, AD, and RD values (ICC:0.90–0.99; *p* < 0.01) at both anatomical sites. The FA value was excellent for the femur (ICC:0.96; *p* < 0.01) and moderate for the tibia (ICC:0.64; *p* < 0.01). The ICC was moderate for the number of tracts in both the femur (ICC:0.72; *p* < 0.02) and tibia (ICC:0.51; *p* < 0.05). There was good inter‐observer agreement regarding the tract volume in the femur (ICC:0.81; *p* < 0.01); however, no significant correlation was seen in the tibia. Tract length showed poor and insignificant inter‐observer agreement.

Pearson's correlation coefficient (*R*) for all the different variables was significant in both the femur (Table 3) and tibia (Table 4). A negative correlation was observed between age and DTI values (tract length, volume, MD, AD, and RD), except for the FA value, which increased over time at both anatomical sites.

**TABLE 3:** Pearson' correlation coefficient of the femur regarding age and different variables from the DTI assessment

	Age	Tract number	Tract volume	FA value	MD	AD	RD
Age	1.000	−0.767 *p* < 0.001	−0.721 *p* < 0.001	0.555 *p* < 0.01	−0.828 *p* < 0.001	−0.818 *p* < 0.001	−0.825 *p* < 0.001
Tract number		1.000	0.976 *p* < 0.001	−0.769 *p* < 0.001	0.821 *p* < 0.001	0.780 *p* < 0.001	0.837 *p* < 0.001
Tract volume			1.000	−0.814 *p* < 0.001	0.765 *p* < 0.001	0.705 *p* < 0.001	0.794 *p* < 0.001
FA value				1.000	−0.718 *p* < 0.01	−0.616 *p* < 0.001	−0.771 *p* < 0.001
MD					1.000	0.990 *p* < 0.001	0.996 *p* < 0.001
AD						1.000	0.974 *p* < 0.001
RD							1.000

*Note*: The correlation is measured in *R*.

**TABLE 4:** Pearson' correlation coefficient of the tibia regarding age and different variables from the DTI assessment

	Age	Tract number	Tract volume	FA value	MD	AD	RD
Age	1.000	−0.722 *p* < 0.001	−0.621 *p* < 0.001	0.730 *p* < 0.001	−0.703 *p* < 0.001	−0.596 *p* < 0.01	−0.728 *p* < 0.001
Tract number		1.000	0.901 *p* < 0.001	−0.900 *p* < 0.001	0.882 *p* < 0.001	0.790 *p* < 0.001	0.901 *p* < 0.001
Tract volume			1.000	−0.856 *p* < 0.001	0.742 *p* < 0.001	0.624 *p* < 0.001	0.772 *p* < 0.001
FA value				1.000	−0.839 *p* < 0.001	−0.630 *p* < 0.001	−0.875 *p* < 0.001
MD					1.000	0.998 *p* < 0.001	0.997 *p* < 0.001
AD						1.000	0.972 *p* < 0.001
RD							1.000

*Note*: The correlation is measured in *R*.

## 4 DISCUSSION

This is the first paper to demonstrate the assessment of a growth plate stratified by age using MRI‐DTI, μCT, and histology. Correlations between the different modalities were significant, and DTI provided additional information about the growth potential of the physes.

We used a multi‐gradient echo 3D sequence to assess growth plate height and volume and found that it performed equal to the other modalities. Our study supports the findings of Wada et al. who used Japanese white rabbits to evaluate the tibial growth plate after physeal injuries using PD‐weighted sequences.^27^ Additionally, we observed a correlation of height measurements between MRI using multi‐gradient echo 3D sequence and histomorphometry. Moreover, our study reports a similar correlation with μCT. The correlation between the modalities is of interest because previous MRI studies have shown different results in assessing the growth plate, depending on whether the assessment has been performed on a cartilage sequence or T1‐weighted sequence.^28^ Our study strengthens the hypothesis that the multi‐gradient echo 3D sequence accurately reproduces the growth plate on MRI.

New Zealand white rabbits reach puberty around day 150 (150/7 = 21.4 wk). One rabbit day during puberty corresponds to 28 human days.^25^ Kilborn and Uhthoff found that both the femoral and tibial growth plates in New Zealand white rabbits closed around the age of puberty and that the femoral growth plate may even close prior to sexual maturation.^4^ The Wilcoxon signed‐rank test demonstrated that the DTI metrics, as well as the tractography, indicated earlier closure of the femoral growth plate in comparison to the tibial growth plate. A deeper inspection of the multi‐gradient echo 3D sequence showed that two of our 24‐wk‐old rabbits had completely fused growth plates in the femur, while the tibia had minor remnants of a high signal at the edges of the growth plate (Figure 4). Similarly, the high‐resolution histological images showed a small chondrocyte remnant of the growth plate in the rabbits that was completely fused. A growth plate remnant has also been reported in human subjects.^28^, ^29^, ^30^ Our findings indicate a difference between chronological and biological ages. Kaweblum et al.^31^ showed that the growth plate fused earlier in New Zealand white rabbits in the femur (radiograph: 20–23 wk; histology:19–24 wk) than in the tibia (radiograph: 22–27 wk; histology: 25–32 wk). Our findings support that New Zealand white rabbits present a suitable animal model for humans regarding skeletal maturity, and that the age group selected in this study resembles adolescent and young adult human females.

The brush‐like appearance of the transitional zone between the growth plate and the metaphysis seen on μCT raises the questions if this is what we see on tractography. Previous studies have demonstrated that the height of the hypertrophic and proliferative zones increases during periods of increased growth velocity and that linear growth can be expressed as Growth *= N* * *h*_max_ ^32^ (Growth is the 24 h growth in μm; *N* is the number of chondrocytes created each day in the proliferative zone; *h*_max_ is the average height of fully mature chondrocytes [μm] in the hypertrophic zone). Similar patterns in the articular and physeal cartilage have been described in experimental DTI studies in rats^19^, ^20^ as well in human cartilage.^33^ The latter study showed that when DTI was performed perpendicular to the transitional zone, type II collagen appeared sideways in the central portion of the articular cartilage. However, in the transitional zone, it was seen parallel to the chondrocyte columns between cartilage and bone, thus demonstrating directional diffusion. We believe that these observations are key to proving the hypothesis that growth velocity can be measured by DTI.

Tractography and DTI metrics showed the same trends in both the femoral and tibial growth plates regarding tract number, volume, MD, AD, and RD. The tibial FA value increased over time, while the femoral FA value reached a plateau. We believe that this finding is a result of earlier closure of the femoral growth plate compared to that of the tibial plate. The MD, AD, and RD values decreased with age and had strong (*p* < 0.001) correlations with each other, indicating that they were interchangeable. Diffusion tractography is sensitive to the *b*‐value, but it has been demonstrated that both DTI metrics and tractography seem to reach a plateau when the b‐value is higher than 750 s/mm^2^.^19^ Therefore, we considered the *b*‐value in our study to be adequate.

DTI depicts the symmetrical pattern created by chondrocytes and supporting structures in the growth plate rather than the chondrocytes themselves. Our results show that, in addition to an age‐related decline of tract number and volume, we also detected tracts extending into the metaphysis in two 24‐wk‐old rabbits that had completely closed growth plates. We agree that tract volume may be associated with growth velocity; however, it is important to remember that the tracts are mathematical constructs and do not represent the corresponding tissue, that is, the growth plate cartilage.^14^ These supporting structures should be considered when assessing growth plates in the later stages of maturity.

The gradient field is spatially dependent, and diffusivity varies depending on whether the measurement is made in the iso‐center, which could have affected our measurements (FA, MD, AD, and RD). In our study, the MRI images were acquired simultaneously for both hind limbs (four growth plates); therefore, every growth plate had a different positioning (all, to some extent, off‐center). Clinical systems' correction technique software can adjust for positioning, but even with this software, the apparent diffusion coefficient ratio error ranges from 2.8% to >10%.^34^ Our preclinical 9.4T system does not have such software, which might have affected the diffusivity. Further, diffusivity is dependent on the temperature of a tissue, which significantly decreases at lower temperatures.^35^ All our test subjects were imaged at a temperature of 25°C instead of body temperature. This is because imaging was performed after euthanization, and the hind limbs were separated from the body prior to imaging. The lower temperature probably lowered the diffusivity; however, we did not observe any motion artifacts, which improved the image quality. The effect of gradient nonlinearities on diffusion MR has been reviewed by Mesri et al.,^36^ who stated that it can result in false outcomes and conclusions. We feel confident that our DTI metrics are adequate because we did not observe a wide variety of FA, MD, AD, and RD values without distinct outliers in our results.

The technical parameters in our study were set to be clinically applicable to pediatric patients. Therefore, we did have larger voxels and fewer diffusion gradient directions than those in other animal studies. A reduction in voxel size with the same field of view and signal‐to‐noise ratio would increase the acquisition time because the signal‐to‐noise ratio is proportional to voxel size and √NEX (Scan time: pulse repetition time x number of phase encoding steps [PE] x number of excitations [NEX]). Similarly, more diffusion gradient directions and higher *b*‐value would also increase the time of acquisition. It has been documented that the FA value does not change so much when the number of directions is more than 14. On the other hand, the tract length and volume are strongly dependent on the number of directions, especially when they are lower than 40.^20^ The number of directions might explain why the ICC was good‐to‐excellent for the DTI metrics (FA, MD, AD, and RD) and lower for tractography (tract number, volume, and tract length). It is noteworthy that with our technical settings, we still see visible tracts and measurable DTI metrics. This further strengthens the thesis that DTI can detect tracts in clinical patients with even larger voxels and lower *b*‐values than that previously reported.^17^, ^20^, ^21^, ^22^, ^37^

### 4.1 Limitations

The resolution of MRI and μCT may have affected the measurements, especially considering the partial volume effect. Wang et al. stated that the orientation variation of collagen in the cartilage was traceable at 90 μm spatial resolution, and fiber orientation in the transitional zone can be resolved at 45 μm spatial resolution.^19^ Manual tracing on a pixelated image can negatively affect observer agreement. Our study was ex vivo, and there is some uncertainty as to whether this would have affected the DTI measurements. Moreover, the number of animals included in the study was low; therefore, a study with a larger cohort and wider age spectrum would be valuable.

## 5 CONCLUSIONS

To the best of our knowledge, this is the first study to visualize the growth plate using μCT, MRI‐DTI, and histology at different ages. This study validates the use of MRI as a non‐ionizing and non‐invasive diagnostic tool to evaluate the growth plate in clinical settings, with no significant difference compared with histomorphometry and μCT. MRI‐DTI can be used as a tool to indirectly visualize the activity and growth potential of the growth plate, although this method is still under development. Furthermore, we believe that the combination of μCT and histomorphometry confirms that DTI depicts the area of the growth plate involved in osteogenesis, that is, growth velocity. However, further studies in both humans and animals are required to validate these results.
